# Structural Analysis of Mitochondrial Mutations Reveals a Role for Bigenomic Protein Interactions in Human Disease

**DOI:** 10.1371/journal.pone.0069003

**Published:** 2013-07-09

**Authors:** Rhiannon E. Lloyd, John E. McGeehan

**Affiliations:** 1 Cellular and Molecular Neuro-Oncology Group, Institute of Biomedical and Biomolecular Sciences, School of Pharmacy and Biomedical Sciences, University of Portsmouth, Portsmouth, United Kingdom; 2 Biophysics Laboratories, Institute of Biomedical and Biomolecular Science, School of Biological Sciences, University of Portsmouth, Portsmouth, United Kingdom; University of Texas Health Science Center at San Antonio, United States of America

## Abstract

Mitochondria are the energy producing organelles of the cell, and mutations within their genome can cause numerous and often severe human diseases. At the heart of every mitochondrion is a set of five large multi-protein machines collectively known as the mitochondrial respiratory chain (MRC). This cellular machinery is central to several processes important for maintaining homeostasis within cells, including the production of ATP. The MRC is unique due to the bigenomic origin of its interacting proteins, which are encoded in the nucleus and mitochondria. It is this, in combination with the sheer number of protein-protein interactions that occur both within and between the MRC complexes, which makes the prediction of function and pathological outcome from primary sequence mutation data extremely challenging. Here we demonstrate how 3D structural analysis can be employed to predict the functional importance of mutations in mtDNA protein-coding genes. We mined the MITOMAP database and, utilizing the latest structural data, classified mutation sites based on their location within the MRC complexes III and IV. Using this approach, four structural classes of mutation were identified, including one underexplored class that interferes with nuclear-mitochondrial protein interactions. We demonstrate that this class currently eludes existing predictive approaches that do not take into account the quaternary structural organization inherent within and between the MRC complexes. The systematic and detailed structural analysis of disease-associated mutations in the mitochondrial Complex III and IV genes significantly enhances the predictive power of existing approaches and our understanding of how such mutations contribute to various pathologies. Given the general lack of any successful therapeutic approaches for disorders of the MRC, these findings may inform the development of new diagnostic and prognostic biomarkers, as well as new drugs and targets for gene therapy.

## Introduction

**Figure 1:**
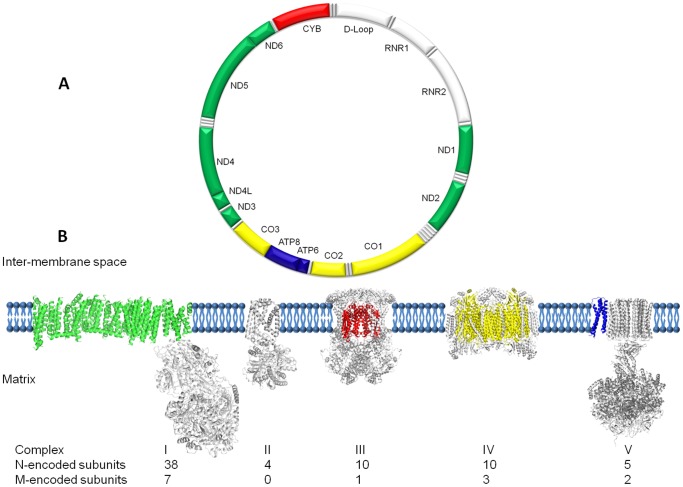
Architecture of the mitochondrial genome and respiratory chain. (A) Schematic representation of the 16,569 bp human mitochondrial genome (NC_012920), with the protein-coding genes colored according to the complexes to which they contribute subunits, two ribosomal RNAs, 22 tRNAs and non-coding D-loop in white. (B) Montage depicting the structural information currently available for the five complexes that together contribute to the mitochondrial oxidative phosphorylation machinery. Each complex (to scale) is embedded in a cartoon representation of the lipid bilayer with the mitochondrial (m)-encoded subunits colored corresponding to the genome diagram. The nuclear (n)-encoded subunits are shown in grey with the relative contributions found in higher organisms detailed below.

Mitochondria are double-membrane, energy-producing organelles in eukaryotic cells and contain multiple copies of their own genome; mitochondrial DNA (mtDNA) (Figure 1A). The apparatus that mitochondria use to generate the majority of cellular energy is the mitochondrial respiratory chain (MRC) located in the inner membrane. Recent publication of the entire Complex I [1], [2] completes the set of crystal structures and examples for all five multi-subunit protein complexes that make up the MRC are now available (Figure 1B). Complexes I to IV transport electrons from NADH and FADH_2_ to molecular oxygen in the mitochondrial matrix. This provides sufficient energy for Complex I, III and IV to translocate protons from the matrix to the intermembrane space, generating a proton gradient. The flow of protons back across the inner mitochondrial membrane to the matrix then drives Complex V to synthesize ATP from ADP and inorganic phosphate [3]. Many cellular processes are coupled to the electron flow through the MRC, including regulation of nucleotide pools, tricarboxylic acid-cycle flux, one-carbon metabolism and reactive oxygen species (ROS) signaling (reviewed in [4]). Additionally, several processes are coupled to the proton motive force, a consequence of the proton gradient generated across the MRC, including calcium transport, NADPH generation, ATP/ADP exchange, protein import, inorganic phosphate transport and mitochondrial membrane potential (reviewed in [4]). Disruptions in the MRC therefore have significant capacity to affect cellular homeostasis and phenotype.

The MRC is unique in the cell due to the bigenomic origin of its protein subunits. Nuclear DNA (nDNA) contributes some 70 subunits and mtDNA contributes the remaining 13 subunits to the MRC (Figure 1A&B). The mitochondrial-encoded proteins are found within the membrane-spanning domains of each complex and perform the principal functions of the MRC. These core subunits contain a variety of redox centers and represent ancient machinery that has remained relatively unchanged from its prokaryotic origins. However, the mammalian cell environment offers significant challenges for a regulated energy-producing system based on electron transfer. The evolutionary consequence, particularly for Complex III and IV, is the synthesis of an elaborate multi-subunit protein shell that protects the core from oxidative damage while endowing it with exquisite control over catalytic activity. The ordered biogenesis and assembly of these structures involves a multitude of accessory proteins necessary for orchestrated transcription, processing, translation, chaperoning and assembly [5]. We focus this study on Complexes III and IV since the availability of closely homologous high-resolution bovine structures provides an excellent template for detailed mapping analysis (Table 1). Furthermore, in addition to their central role in the MRC, these two complexes represent paradigms of quaternary protein organization and reveal the intimate relationship between mitochondrial and nuclear-encoded subunits.

**Table 1:** Conservation of mitochondrial-encoded subunits within the complexes (I–V) between human and its high resolution structural homologs.

Complex	Structural Homolog (PDB No, Ref)	Chain (s)	*H. sapiens* (mt-)	Homology (%)
I	*T. thermophilus* (4HEA and 3M9S, [2], [70])	H, T	ND1	41
	*E. coli* (3RKO, [1])	D, N	ND2	21
		A, E	ND3	28
		C, M	ND4	28
		G, K	ND4L	22
		B, L	ND5	27
		F, J	ND6	12
III	*B. taurus* (1NTZ, [10])	C	CYB	78
IV	*B. taurus* (2EIJ, [40])	A, N	CO1	91
		B, O	CO2	75
		C, P	CO3	88
V	*E. coli* (1C17, [73])	M	ATP6^a^	23

a There is no high resolution structural homolog for the mitochondrial-encoded ATP8 present in humans.

Complex III (EC 1.10.2.2) occupies the middle position of the MRC and couples the transfer of electrons from ubihydroquinone to cytochrome *c* while generating a proton gradient across the inner mitochondrial membrane [6], [7]. Complex III, which is also known as cytochrome *bc*_1_ (Cyt *bc*_1_) and ubiquinol: cytochrome *c* oxidoreductase, is a dimer where each monomer is composed of 11 different subunits [8]. There are three redox-active subunits in each half; the iron-sulphur protein (ISP), cytochrome *c_1_* and the only mitochondrial-encoded subunit, cytochrome *b*, located centrally in the trans-membrane region (Figure 1, gene and protein depicted in red). These subunits harbor a Rieske-type Fe_2_S_2_ iron-sulphur cluster and two hemes, *b*_H_ and *b*_L_ which together facilitate the proton-motive Q-cycle mechanism [9]. There are two separate quinone binding sites, the Q_o_ site for quinone oxidation and the Q_i_ site for reduction. There is a bifurcated electron flow at the Q_o_ site where two electrons are transferred from ubihydroquinone to two different chains. The first electron is transferred to the ISP, then onwards to cytochrome *c*_1_ and finally to cytochrome *c*. The second electron is transferred sequentially to heme *b*_L_, then *b*_H_, and onward to the ubiquinone bound at the Q_i_ site. Two protons are released from the intermembrane space for every quinol oxidised. In the full catalytic cycle, two molecules of quinol are consumed at the Q_o_ site, one quinol molecule is generated at the Q_i_ site, and a total of four protons are translocated across the mitochondrial membrane from the matrix to the intermembrane space [10].

Complex IV (EC 1.9.3.1), or cytochrome *c* oxidase (COX), is the terminal component of the MRC, which reduces molecular oxygen to water with the electrons from the reduced form of cytochrome *c*, a product of Complex III, while pumping protons from the mitochondrial matrix to the intermembrane space [11], [12]. Complex IV has three main areas: an outer region that faces the inter-membrane space, an inner region that faces the matrix side and a large trans-membrane region. The complex is composed of 13 subunits: three mitochondrial-encoded subunits that form the central core (CO1, CO2 and CO3: depicted in yellow in Figure 1B) and 10 nuclear-encoded subunits that form a structural scaffold around the core [13]. The complex also contains two iron sites (heme *a* and *a*_3_), two copper sites (Cu_A_ and Cu_B_), as well as zinc and magnesium sites [14]. Electron and proton transfers are performed by the mitochondrial-encoded CO1 and CO2 subunits. Mitochondrial-encoded CO1 and CO2 also create the channels required for the dioxygen molecule to reach, and the H_2_O molecule to be removed from, the O_2_ reduction site. In the complete cycle, four electrons are transferred to molecular oxygen (O_2_) from four molecules of cytochrome *c* producing two molecules of water. A total of four protons are removed from the mitochondrial matrix of which two are translocated across the membrane. In combination with Complex I, Complexes III and IV maintain the proton gradient that is the driving force behind ATP-generation in the MRC via the F_O_F_1_ ATP synthase [15], [16].

Mutations in the genes that encode protein components of the MRC are associated with numerous diseases, ranging from classical mitochondrial disorders (e.g. Leber’s hereditary optic neuropathy (LHON), Mitochondrial Encephalomyopathy, Lactic Acidosis, and Stroke-like episodes and Leigh syndrome [17]) to more common disorders, including neurodegenerative diseases (e.g. Parkinson’s [18]), cancers (e.g. prostate [19]) and metabolic diseases (e.g. obesity [20]). Large scale genomic projects, including the Cancer Genome Atlas (TCGA), are opening up new possibilities in the development of personalized medicine [21]. Both maternally inherited and somatic mtDNA-mutations are increasingly being discovered, and a recent report has revealed the spectrum of mitochondrial mutations in five cancers following the whole-genome massively parallel sequencing of tumor and non-tumor tissue pairs [22]. However, it is not always clear which mitochondrial mutations are functionally relevant and contribute to disease due to several confounding factors: 1) multiple mtDNA molecules are resident in affected tissues and only a proportion of these are mutated (a state referred to as heteroplasmy) [23], 2) the number of mutant mtDNAs must exceed a certain threshold for a functional effect to be observed, 3) the threshold at which a phenotype is observed varies between tissues [24], [25] and 4) often multiple nuclear and mitochondrial mutations are present [22].

One way to identify whether a mtDNA mutation is pathogenic is to demonstrate that mitochondria harboring it, either in patient samples or transmitochondrial cybrids, have a corresponding biochemical defect [26], however, it is simply not possible to do this for every mutation; thus, their role in pathogenicity remains unresolved. Computer-based algorithms can be used to evaluate the affect of amino acid substitutions (AAS) that result from mutations in mtDNA on protein function *in silico* and an integrated approach using the SIFT (http://sift.jcvi.org) and PolyPhen (http://genetics.bwh.harvard.edu/pph2) programs has recently been published [27]. Similarly, MutationAssessor has recently been used to predict the functional impact of mitochondrial AAS in cancer [22].

SIFT and MutationAssessor are sequence homology-based approaches and both use multiple alignments of homologous and distantly related sequences to identify which positions are evolutionarily conserved within and between species. If the AAS is conserved across multiple sequences it will be deemed as important for protein function. Polyphen uses sequence homology together with a combination of extracted data, such as protein function annotations from the UniProtKB database, secondary structure information from the DSSP program [28], [29], crystallographic B-factors from the RCSB Protein Databank (PDB) [30] and calculated structural parameters including accessible surface area, hydrophobicity and electrostatics, to arrive at a prediction. If the alignments, annotations or basic structural parameters suggest the AAS is conserved, buried in the protein structure or found within an active site, a ligand-binding region, is part of a disulfide bridge or a protein-protein interaction region, it will be predicted as pathogenic.

Despite their predictive potential, these generic algorithms can perform sub-optimally and yield contradictory information when applied to the MRC. One reason is the lack of any human MRC protein complex structures. All three programs deal with this by utilizing sequences from lower organisms for the analysis of sequence variation. Given the potential structural and functional differences between the human and distantly related template proteins, it is possible that the role of specific AAS can be missed or misinterpreted. Although the main features of the catalytic subunits are often widely conserved, the situation in the eukaryotic cell is often more complicated, particularly where multiple additional accessory subunits generate new interfaces. This is particularly relevant when sequence information from bacterial structural homologues of Complex I and V are used.

We therefore employed 3D structural analysis, more appropriate for the bigenomic, quaternary structural organization of the MRC, to determine the functional importance of AAS in Complex III and IV mitochondrial proteins. We demonstrate how this approach can be used for predicting pathogenicity and shedding-light on disease mechanisms. Additionally, we identify four structural classes, including an elusive and as yet underexplored class of mitochondrial mutations that affect the assembly and stability of the complexes studied. The work presented here provides an extension to current methods, reveals novel interactions that can underpin specific mitochondrial diseases and will benefit those in the field that wish to predict clinical outcomes from the wealth of emerging genetic information.

## Results and Discussion

### The Development of a Predictive Approach

Current available tools are extremely useful, but often yield contradictory information when applied to mitochondrial mutations, falling short of the Holy Grail which is to directly inform the clinician of the likelihood and severity of mitochondrial syndromes and diseases from the results of a straightforward genetic test. This remains challenging due to the complex architecture of the protein complexes that are encoded by the mitochondrial genome, as shown in Figure 1, but also because there is significant cross-talk with an array of nuclear-encoded proteins. Nevertheless, we find ourselves in a fortunate position where there is a large collated database of mitochondrial mutations publically available, a vast body of literature that includes everything from patient data to detailed biochemistry, and a complete set of MRC protein complex crystal structures, albeit from human orthologs.

**Figure 2:**
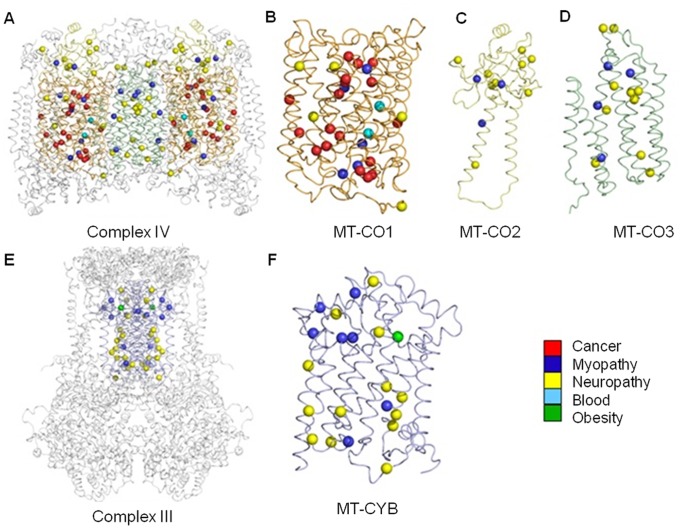
A new structural map of human mitochondrial disease mutation sites in Complexes III and IV. Mitochondrial diseases result in diverse pathology, and can be multi-systemic or tissue-specific. Here we show 93 individual mutation sites mapped onto their corresponding 3D crystal structures. Each mutation site is shown as a sphere colored according to the primary pathology or tissue (see legend) found to be affected in either single or groups of patients. The complete dimeric Complex IV is depicted as a ribbon model (A) with the mitochondrial-encoded subunits, MT-CO1 (B), MT-CO2 (C) and MT-CO3 (D), colored orange, yellow and green, respectively. The three COX monomers are shown separately for clarity. The dimeric Complex III is shown in the same format (E) with the mitochondrial-encoded MT-CYB (F) subunits colored as blue ribbons.

Our approach, summarized in Figure S2, involved taking all 13 human mitochondrial protein sequences to find the most homologous protein structures currently available and this allowed us to compile Figure 1B. We chose to model AAS in proteins that, following alignment, were at least 60% identical between the human sequences and the homologs identified, as this has been shown to improve prediction accuracy [31]. For the time being, this restricted the application of the 3D structural analysis to mutations in mtDNA genes that encode the protein subunits for the structurally and biochemically well-defined bovine complexes III and IV of the MRC (Table 1 and Figure S1). Nonetheless, as more complete MRC structural homologues become available, it will become possible to predict the pathogenicity of the mutations in the remaining 9 mtDNA genes, increasing the predictive power of our approach. In parallel, the MITOMAP database was consulted for published mutations with disease-associations and all 93 found in Complex III and IV genes were mapped onto their bovine protein structural homologs (Figure 2). Direct biochemical evidence from the literature was then collated to formulate a set of mutations we call ‘Confirmed mutations’ to test our approach (Table 2). Patient samples from this set have biochemical data that confirm that the identified mutation causes dysfunction of the associated protein complex in the MRC. In some cases, this is supported by the availability of cybrid data: that is, where a cell line containing that particular mutation has been generated and analyzed. The set of ‘Confirmed mutations’ represents the current best link available between genotype and phenotype, and it is these we analysed further and used as the basis to define four structural classes of mutations. Subsequently, we have used the approach to assess ‘Unconfirmed mutations’: those associated with a disease, but without supporting biochemical evidence (Table 3). The final goal of this exercise is to allow the pathogenicity of any novel mutation in Complex III and IV genes to be assessed from the individual patient sequence data alone (Figure S2).

**Table 2:** Structural classification and pathogenic prediction of mutations in the mtDNA complex III and IV genes (mt-*cyb* and mt-*co1-3*, respectively) reported with known human disease associations and biochemical effects.

					Percentage Complex Activity										Predictions			
Human subunit(Gene)	Human residue (Nucleotide)	Human disease	Patient Sample	O_2_ Consu-mption	I	II	III	I+III	II+III	IV	V	Ref	Class	Hetero−/Homo-plasmy	SIFT	Polyphen	MA	3D Modeling
MT-CYB (*mt-cyb*)	Q352Ter (C15800T)	EXIT/Myopathy	Muscle		−71	ND	−69			ND	ND	[78]	1	Het				Pathogenic
	G105Ter (G15059A)	Mitochondrial Myopathy	Muscle					−89	−85			[79]	1	Het				Pathogenic
	G34S (G14846A)$	EXIT	Muscle		−23	4		−80	−79	−15		[80]	3	Het	Affect	Pathogenic	High	Pathogenic
	S35P (T14849C)	Septo-Optic Dysplasia	Muscle		−13	−15	−49			−24		[45]	3	Het	Affect	Pathogenic	High	Pathogenic
	Y278C (A15579G)	Multisystem Disorder	Muscle		−11	−25	−94			3		[46]	3	Het	Affect	Pathogenic	High	Pathogenic
	G339E (G15762A)	Mitochondrial Myopathy	Muscle					−89	−75			[47]	3	Het	Affect	Pathogenic	High	Pathogenic
MT-CO1 (*mt-co1*)	AELGQ39AGPA Ter (6020del5)	Motor Neurone Disease	Muscle		18	−11		−6	−1	−57		[35]	1	Het				Pathogenic
	M273T (T6721C)$	AIS-Anaemia	Cybrid	−30	ND		ND			ND		[38]	2	Het	Affect	Pathogenic	High	Pathogenic
	I280T (T6742C)$	AIS-Anaemia	Cybrid	ND	ND		−25			−38		[38]	2	Het	Affect	Pathogenic	High	Pathogenic
	G352D (G6955A)	Mild EXIT and Mental Retardation	Muscle			−31	−70	−77	−79	−89		[42]	2	Het/Homo	Affect	Pathogenic	High	Pathogenic
			Fibroblast						−2	−39								
MT-CO2(*mt-co2*)	M29K (T7671A)	Mitochondrial Myopathy	Muscle		245				157	−75		[57]	4	Het	Affect	Pathogenic	High	Pathogenic
MT-CO3 (*mt-co3*)	Q111Frameshift (C9537insC)$	Leigh-like syndrome	Muscle		17	17	26			−89	37	[81]	1	Homo				Pathogenic
			Fibroblasts		−39	−29	−12			−78	4							
			Cybrid		−1		−31			−89	−19							

Abbreviations: Percentage – O_2_ consumption and mitochondrial complex activities are expressed as percentages relative to published mean control values, further details can be found in the materials and methods, MA – MutationAssessor, EXIT - Exercise Intolerance, ND – No Difference, AIS – Acquired Idiopathic Sideroblastic,

$ reported as somatic (rather than inherited) mutation, Het – reported as a mixture of wild-type and mutated mtDNAs in affected patients, Hom – reported as mutated mtDNAs only in patients, Het/Homo – reported as heteroplasmy in some patients but or homoplasmy in others.

**Table 3:** Structural classification and pathogenic prediction of mutations in the mtDNA complex III and IV genes (*mt-cyb and mt-co1-3*, respectively) reported with human disease associations but without known biochemical effects.

						Predictions			
Human subunit (Gene)	Human residue (Nucleotide)	Human disease	Reference	Hetero−/Homo-plasmy	Class	SIFT	Polyphen	Mutation Assessor	3D Modeling
MT-CYB(*mt-cyb*)	N32S (A14841G)	LHON helper mutation	[82]	Het	3	Affect	Pathogenic	High	Pathogenic
MT-CO1(*mt-co1*)	V374M (G7023A)	MELAS-like syndrome	[83]	Het	2	Affect	Pathogenic	High	Pathogenic
	V380I (G7041A)*	Prostate Cancer	[84]	Homo	2	Affect	Pathogenic	High	Pathogenic
	A120T (G6261A)*	Prostate Cancer/LHON	[58], [59], [84]	Homo	4	Tolerated	Benign	Neutral	Pathogenic
	I419V (A7158G)*	Prostate Cancer	[19]	Homo	4	Tolerated	Benign	Neutral	Pathogenic
	V193I (G6480A)*	Prostate Cancer	[19], [20], [85]	Homo	4	Tolerated	Benign	Neutral	Pathogenic
	M117T (T6253C)*	Prostate Cancer	[19], [58]	Homo	4	Tolerated	Benign	Medium	Pathogenic
MT-CO2(*mt-co2*)	E18K (G7637A)*	PD risk factor	[86]	Het/Homo	4	Affect	Pathogenic	High	Yes
	D92N (G7859A)*	Progressive Encephalomyopathy	[87]	Homo	4	Tolerated	Benign	Neutral	Pathogenic
	L95F (C7868T)	LHON	[82]	Homo	4	Affect	Benign	Medium	Pathogenic

Abbreviations: LHON – Leber Hereditary Optic Neuropathy,

* also reported as polymorphism,

$ reported as somatic (rather than inherited) mutation, Het – reported as a mixture of wild-type and mutated mtDNAs in affected patients, Hom – reported as mutated mtDNAs only in patients, Het/Homo – reported as heteroplasmy in some patients but or homoplasmy in others.

### A Comprehensive Structural Map of Complex III and IV Mitochondrial Disease Mutations

We revealed that a wide range of pathologies are associated with mitochondrial-encoded core protein mutation sites of Complexes IV and III (Figure 2). This is the first visual map, to our knowledge, of mutation sites found in either single or groups of patients in mitochondrial proteins. On initial inspection, we reveal the mutation sites are widely distributed around the core of Complex IV and Complex III. On closer inspection, more mutation sites are present in MT-CO1 than the other three proteins (MT-CO1-2 and MT-CYB). While prostate cancer and blood disorder mutation sites are specific to MT-CO1, and the only common metabolic disorder mutation site is specific to MT-CYB, myopathy and neuropathy mutation sites are present in all Complex III and Complex IV mitochondrial core proteins. Mutation sites are also apparent that tend to localize in specific regions of the subunits. With the sequencing of whole genomes from affected tissues in individual patients becoming more common place, there is now an opportunity to build this map further and develop our understanding of which protein-coding genes are more susceptible to mutation and whether certain clusters of mutations are more likely to be associated with either the same, different or even multiple disease states. It is also becoming evident from whole genomic studies that affected tissue from individual patients, for example those with glioblastoma multiforme, often harbor multiple mitochondrial mutations [22]. There is now a possibility of building structural maps that will enable us to visualize and predict how functional AAS in mitochondrial proteins interact within Complexes III and IV and contribute to metabolic deregulation on a patient-specific basis.

### Systematic Structural Classification as a Platform for Prediction of Function

We have systematically classified the available mutation data for Complex III and IV to simplify a complicated and diverse dataset. Our system takes advantage of current mutation data with associated biochemistry, the so called ‘confirmed’ set. Although some previous studies have presented elegant structure-function examples for individual mutations, such a wide classification, to our knowledge, has not been previously reported. This allowed us to identify new patterns and associations that can in turn be used to predict the functional outcome of those mutations that currently lack biochemical analysis. We have identified four main structural and functional classes, and although there can be interplay between each group; generally, individual mutations fall clearly into a single class. Here we present detailed illustrations of confirmed mutations in each class and demonstrate how they can be used as a basis for predicting pathogenic outcomes and mechanisms of unconfirmed mutations, as well as for providing novel targets for drug design. Detailed illustrations of unconfirmed mutations in each class are also provided. In total, we classified and predicted the pathogenic potential of 12 confirmed and 7 unconfirmed mutations, which are available as a resource in Tables 2 and 3.

### Frameshift (Class 1) Mutations cause Protein Subunit Loss

Several diseases have been associated with mutations that disrupt the register of the triplet coding sequence of the mitochondrial genome (e.g. Chronic Progressive External Ophthalmoplegia [32], Exercise Intolerance [33] and Rhabdomyolysis [34]). Whether an insertion or a deletion, this inevitably results in the generation of a stop codon and premature termination of the amino acid chain. The structural outcome of such mutations can be catastrophic resulting in the loss of entire domains within the protein complex. Figure S3 depicts the structural consequence of the confirmed mutation *6020del5* and how it could contribute to the pathogenicity of Motor Neuron Disease [35]. In the wild type, the MT-CO1 subunit maintains much of the catalytic environment for Complex IV (Figure S3A and C). This mutation results in the change of 4 amino acids and the loss of 90% of this subunit, which occurs in both halves of this dimeric complex (Figure S3B and D), reducing Complex IV activity by almost 60% (Table 2). It is likely that the nuclear subunits that decorate the surface of this subunit will not have sufficient surface area to make stabilizing contacts and will be lost into the membrane. This more general loss of quaternary structure will further compromise the activity of the MRC where associations between the complexes have been demonstrated to be vital for efficient electron transport and for their assembly and stability [36]. This is borne out by associated activity loss in other complexes in the chain (Table 2).

### Active Site (Class 2) Mutations Disrupt the Local Chemical Environment

**Figure 3:**
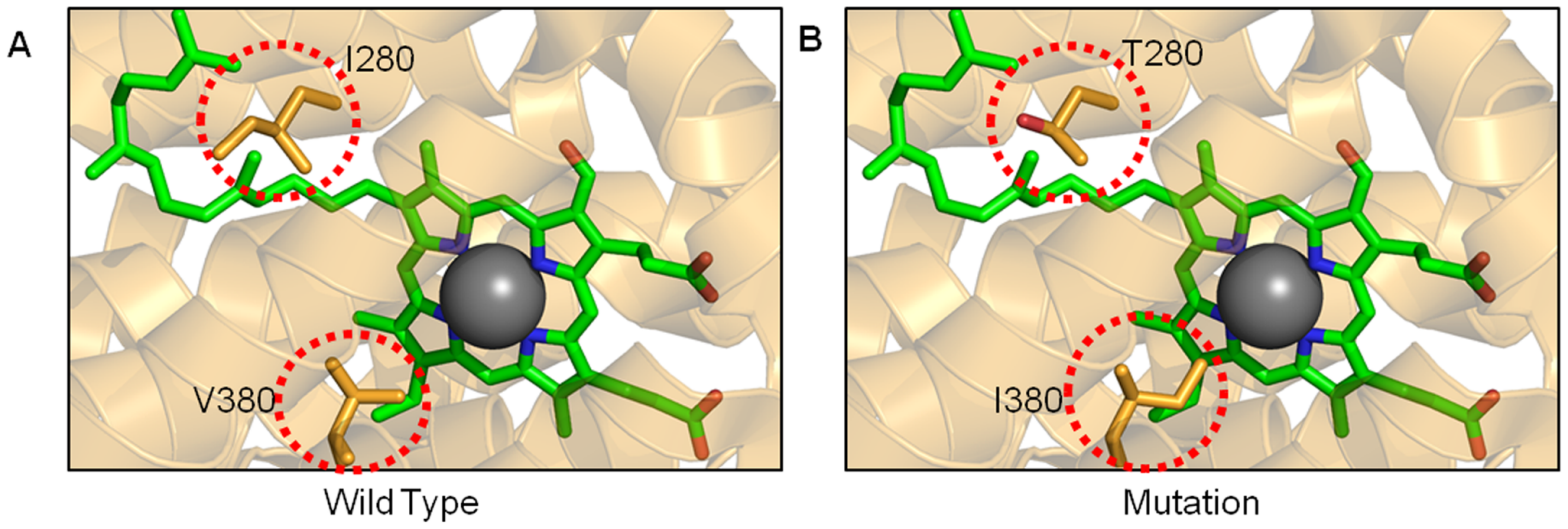
Active site mutations. (A) The active site region of the wild-type MT-CO1 subunit of complex IV is depicted as a ribbon diagram with key amino acids as orange stick representations (within red circles). The heme *a*_3_ is colored in green with the Fe atom in grey. (B) Two separate mutations have been modeled, I280T and V380I. More detailed diagrams can be found in Figure S5.

Complexes I, III, IV and V of the MRC have evolved elegant functional mechanisms to pump protons across the inner mitochondrial membrane by linking multiple substrates with co-factors and metallo-protein sites, while maintaining ROS protection and an efficient environment for local chemistry to take place. While it is well documented that even small perturbations in these active site regions of Complex III and IV have functional consequences (see references in Table 2), Figure 3 illustrates two cases from numerous examples in the database, the first represents a confirmed mutation with associated biochemistry and the second represents an unconfirmed mutation.

Mutation *T6742C* in Complex IV causes the amino-acid substitution I280T and is associated with Acquired Idiopathic Sideroblastic (AIS)-Anemia [37]. Detailed studies of this mutation have been carried out using artificially generated cybrid cell lines that harbor the change [38]. Such studies represent a standard methodology for looking at the effect of mutated mtDNAs on a relevant nuclear background, but one that requires significant endeavor and is simply not practical for all mutations (reviewed [39]). In this case, cybrids that harbor I280T demonstrate a reduction of approximately 40% in Complex IV activity with a concomitant loss of 25% activity in Complex III, again demonstrating the intimate biochemical relationships between complexes of the MRC (Table 2).

On first inspection, it would seem surprising that such a small substitution from I to T, constituting the loss of a methyl and the gain of a hydroxyl group in a very large structure, would have any effect at all. It is only when the active site environment of subunit MT-CO1 in Complex IV is examined that the proximity to the heme group and the contribution to the chemical environment is revealed (Figure 3A&B) [13]. Heme *a*_3_ and Cu_B_ form the O_2_ reduction site in Complex IV and comparison of multiple high-resolution crystal structures indicates that the cavities in the immediate vicinity of this heme undergo conformational change as a function of the oxidation state [40]. In chemical terms, the substitution of the hydrophobic side chain of I280 with an uncharged polar side chain of T280 is likely to destabilize the heme *a*_3_ and surrounding cavities, particularly given the proximity of the hydroxyl group to the tail of heme *a*_3_ (Figure S5A&B) [41]. It is therefore clear how this mutation can cause such a significant and observable functional effect. When considering that this situation can be amplified to a significant proportion of mitochondria in all cells, it becomes apparent how a single mutation can result in metabolic dysfunction and could contribute, in this case, to AIS-Anemia.

We can use this structural information to predict the effect of unconfirmed active site mutations that do not have detailed associated biochemical studies. Figure 3 also shows a second mutation site in the MT-CO1 subunit that, despite being 100 amino acids away in primary sequence terms, maps within the same heme pocket as the previous example. Mutation *G7041A* has been found in patients with prostate cancer [19]. In this case, the substitution from V380I represents only a single carbon atom extension of the side chain (Figure 3A&B). Again, it would seem surprising that this alone could cause such a dramatic functional effect, until we observed that this additional carbon atom in our model creates a direct steric clash with the central Fe *a*_3_ atom of the heme group (Figure S5C and D). This small helical region, referred to as helix X in the wider literature, is known to undergo a conformational change responding to the redox state of the heme. This is in turn linked to the 3D architecture of the water channel near the hydrogen-bond network junction required for efficient proton-pumping [40], [41].

Mapping of two more confirmed mutations (*T6721C* [38] and *G6955A* [42]) to Heme *a*_3_ further underlines that the environment around this catalytic metal is highly sensitive to even sub-angstrom perturbations (Figure S4). We therefore predict that the biochemical outcome of the *G7041A* will be a significant loss of Complex IV activity due to disruption of the catalytic heme and surrounding area. This in turn may contribute to the shifts in energy metabolism from oxidative phosphorylation to glycolysis, often observed in tumor cells, and may play a significant role in the progression of the disease. This example illustrates how the mapping of confirmed mutations in a structural context can be used to inform others that are not obviously linked at the level of primary sequence, or even disease, to aid our understanding of disease mechanisms and develop new research questions. Consistent with our own predictions based on 3D structural analysis, all three standard algorithms predict this and other confirmed and unconfirmed Class 2 mutations as pathogenic (Table 2 and 3). This is due to these Class 2 mutation sites being highly conserved between multiple species. In both examples presented here, I280 and V380, these residues are 100% conserved among the 6 vertebrate and 4 invertebrate MT-CO1 sequences used by SIFT in the integrated approach [27].

### Binding Pockets (Class 3) Mutations could Explain Patient Sensitivity to Substrates and Inhibitors

Recent advances in electron microscopy have revealed the direct association of MRC complexes within the inner mitochondrial membrane [36], [43], [44], however, it is well established that many of the interactions between the complexes are the result of the shuttling and modification of small molecules [4]. Each complex has multiple binding pockets that can accommodate both substrate and product and these cavities can be exquisitely formed to be highly selective. Such properties represent attractive targets to the pharmaceutical industry. Often these pockets are formed in 3D space from multiple regions, chains or subunits, not obviously proximal when relying on 2D sequence alignments. Again, it is the ability to map mutation sites onto high-resolution structures that yields direct insight into the mechanisms of subtle disease associated mutations [8]. Figure S6 and 4 depict in detail two such examples in the ubiquinone (Coenzyme Q) binding pocket (Q_i_ site) of MT-CYB, one confirmed, *T14849C* (S35P), and one unconfirmed, *A14841G* (N32S), associated with Septo-Optic Dysplasia [45] and LHON [21], respectively. The locations of further confirmed binding pocket mutations, one in the Qi site (*G14846A* [33]) and two in the ubiquinol binding pocket (Q_o_ site)(*A15579G* [46] and *G15762A* [47]) are shown in Figure S4.

The intricate cavity of the Q_i_ site, visualized by the surface rendering in Figure S6C, is formed by a cluster of alpha-helices that together generate an access tunnel from the external environment to the internally located heme *b*_H_. The Q_i_ site has a complex role that is optimized to allow efficient reduction and protonation of the ubiquinone substrate. This is achieved by selective substrate binding in a coordination compatible for electron transfer with the heme *b*_H_ while maintaining connection with the mitochondrial matrix for the supply of protons. Such interactions are highly evolved and even subtle changes can drastically alter binding. For example, Antimycin A, a natural Q_i_ site inhibitor, also binds within this cavity with high affinity and competes with ubiquinone [48]. Studies in the highly related complex from *Leishmania tarentolae* have shown that a subtle S35I mutation confers resistance to Antimycin A, clearly demonstrating the contribution of individual amino acids to selective substrate binding within this pocket [49].

In a human patient with Septo-Optic Dysplasia [45], the S35P substitution at the same position can be considered to be more dramatic than the protozoan S35I substitution in terms of amino acid chemistry. It is likely to generate a severe structural perturbation by inserting P35 into a major helix (helix A) that lines the pocket, in addition to the loss of a hydrogen bond to ubiquinone originally reported [45]. Proline is unusual, being a secondary amine with a cyclic structure that results in a conformational rigidity, and cannot easily participate within an alpha helical fold as it disrupts helical hydrogen-bonding and generates steric incompatibility. It is likely that this mutation will disrupt the correct folding of this alpha helix at the point where it forms the integral surface of the binding pocket. The affected region is depicted as the red surface rendering in Figure S6D. This will disturb ubiquinone-mediated electron transfer from heme *b*_H_ and the production of ubiquinol. The observed biochemical outcome is a reduction of Complex III activity by 49% and a significant concomitant drop in the activity of all the other MRC complexes tested (Table 2). Deficiency in ubiquinone or ubiquinol is likely to disrupt the Q-cycle mechanism that involves electron transfer from ubiquinol at the Q_0_ site to Cytochrome *c* and ultimately Complex IV, explaining the deficiency in both Complex III and IV.

**Figure 4:**
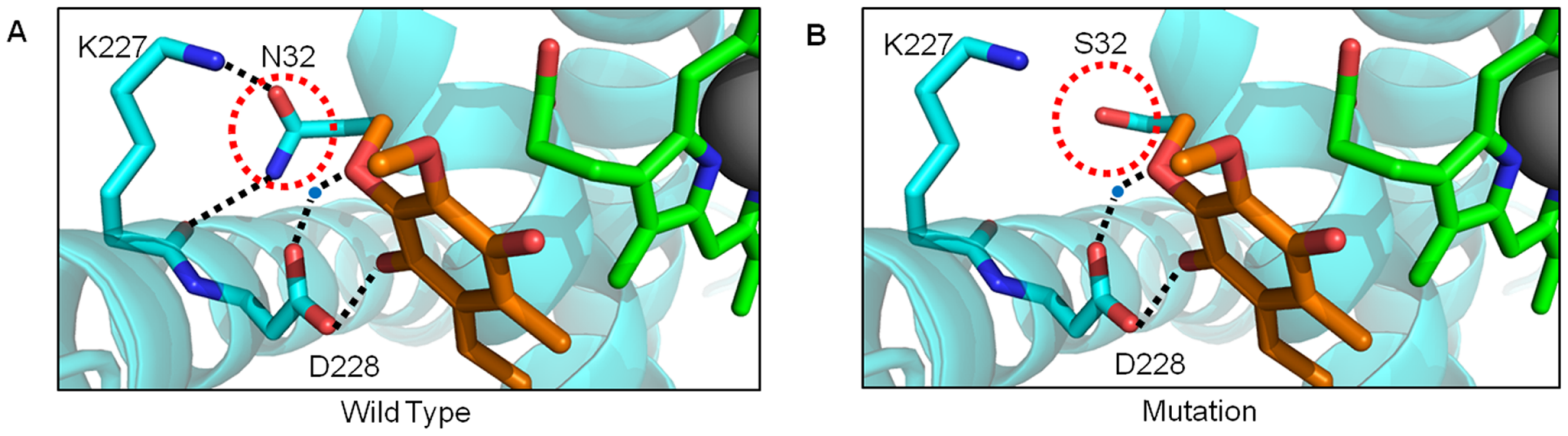
The role of residues contributing to substrate-binding cavities. MT-CYB has several deep pockets for binding, redox interaction and modification of substrates. (A) The Q_i_ site is formed by the contribution of multiple helical regions (blue) that fold around heme *b*_H_ (green) while maintaining contact with the solvent. The wild-type residue N32 (PDB 1NTZ) is shown making hydrogen bonds (dotted lines) with the natural substrate ubiquinone (orange), within the same pocket. (B) Mutation to S32 results in the loss of key hydrogen bonds previously identified to be crucial in the redox mechanism.

The unconfirmed N32S substitution associated with LHON [21] is situated deep within the Qi-pocket, also on transmembrane helix A (Figure 4A&B) and this location alone suggests a role in substrate interactions. Indeed, detailed crystallographic data have revealed three key H-bonds between residues lining the cavity surface in this region (D228, H201 and S205) and the ubiquinone substrate, two of which are water-mediated and thought to be central to the protonation mechanism [10]. Residue N32 participates in stabilizing H-bonds to the main chain and side chain of K227 (Figure 4A). Interestingly, K227 is implicated in the high binding affinity of Antimycin A due to the generation of one of two additional H-bonds compared to the natural substrate ubiquinone. It is precisely these fine details that have the potential to inform drug sensitivities in a patient-specific manner. For example, in this patient it is likely that the N35S substitution would reduce the competitive effect of Antimycin A (Figure 4B).

Residue N32 is also proximal to the side chain of D228 which makes both direct and water-mediated contacts to ubiquinone. It is therefore likely that the mutation N32S will act in a similar manner to the confirmed mutation S35P, disrupting the production of ubiquinol from ubiquinone and the overall Q-cycle mechanism, resulting in significant loss of Complex III function and related detrimental effects to the whole MRC. The predicted ubiquinone deficiency in patients that harbor Q_i_ pocket mutations, such as at P35 and S32 sites, could mean they would benefit from ubiquinone replacement therapy, shown to be effective for known ubiquinone deficiency disorders that present with childhood encephalopathy and seizures, recurrent rhabdomyolysis or ataxia with seizures [50].

Similar to the Class 2 mutations, and consistent with our predictions, all three standard algorithms predict confirmed and unconfirmed Class 3 mutations (Table 2 and 3) to be pathogenic. This is due to the mutation sites occurring at evolutionary conserved residues. Furthermore, unlike the 3D analysis, the standard algorithms do not offer a window into the mechanism of pathogenesis and cannot be used to predict differences in patient sensitivities to inhibitors and substrates. Nor do they allow the fine details of the binding pockets to be visualized in 3D space, providing opportunities for novel drug design. Depending on the mutation(s) present in binding pockets such as the Q_i_ site, novel inhibitors that compete with the natural substrate could be designed to selectively trigger mitochondrially-mediated apoptosis, proposed as a treatment for some cancers including gliomas [51].

### Identification of Novel Nuclear-Mitochondrial Interaction Region (Class 4) Mutations Requires the Detailed Consideration of Quaternary Structure and Subunit Interactions

The 3D structural analysis of disease-associated AAS that result from mutations in mtDNA Complex III and IV protein-coding genes has revealed a previously undefined subset we term Class 4, located at the protein-protein interfaces between the subunits that make up the MRC complexes. Within this class, those that occur at the nuclear-mitochondrial interfaces are the most highly represented within the MITOMAP database. It would seem obvious that the interacting surfaces of the individual proteins that make up the complexes are vital for correct assembly, stability and ultimately function, however, it is these nuclear-mitochondrial interaction region AAS in particular that can remain invisible to current *in silico* pathological predictions. Here we look specifically at Complex IV as no Class 4 mutations were identified in Complex III. Only when new complete MRC structural homologues become available will it become possible to determine whether such mutations also occur in the other bigenomic complexes; I and V.

In Complex IV, denaturation studies employing urea and guanidinium chloride have shown that the individual nuclear subunits are robust and structurally stable, however, their interactions within the complex are much less stable [52]. In particular, 5 of the 13 subunits were shown to be released from the complex under relatively mild denaturing conditions in the order COX6B, COX6A, COX7A, MT-CO3, and finally COX5B. There is now a large body of literature on the role of the individual nuclear-encoded subunits; however, it is experimentally challenging to assign specific function in a cellular context. Subunits including COX4, COX5A, COX5B, and COX6A have been attributed with ATP-dependent regulatory roles [53] and this is borne out by the identification of tissue-specific isoforms of several nuclear-encoded COX subunits [54], [55]. Subunits such as COX6C and COX6B2 appear to have more structural roles since their untimely removal during biogenesis can render the entire complex irreversibly monomeric [56].

**Figure 5:**
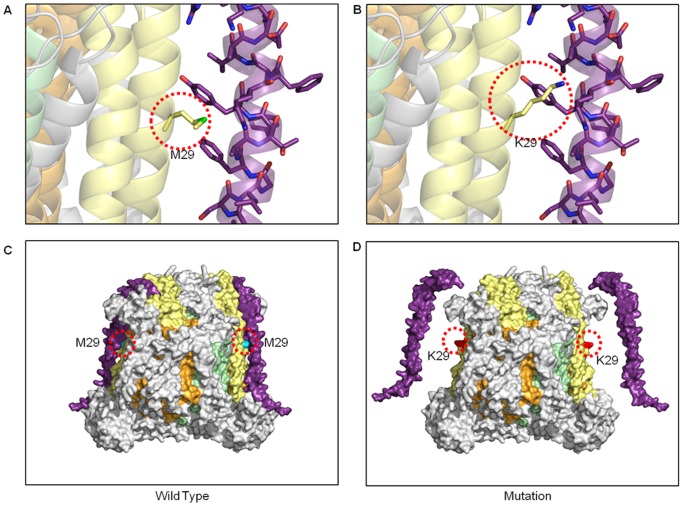
Assembly disruption from bigenomic protein incompatibility in Complex IV. (A) The mitochondrial subunits are surrounded by nuclear-encoded subunits that make intimate interactions, in this case MT-CO2 (yellow ribbons) with COX6C (purple ribbon), respectively. The wild type M29 residue is shown in stick form occupying a position between to aromatic side chains from COX6C. (B) The mutation K29 results in the incorporation of a long and polar side chain that is incompatible within the tight interface between MT-CO2 and COX6C. (C) The wild type-enzyme is shown as a surface model in the same color scheme with the position of the mutation site highlighted by a dotted red circle. (D) A surface illustration depicts the position of the K29 mutation and the potential resulting aberrant assembly of the nuclear subunit COX6C.

Figure 5 illustrates a detailed example where a simple amino acid change results in a sterically incompatible surface between the MT-CO2 and nuclear-encoded COX6C subunits. Despite this mutation site being distal from any of the redox machinery, this is a confirmed AAS and the resulting loss of structural integrity is associated with a 75% loss of Complex IV activity and a myopathy [57] (Table 2). The M29K change on the surface of MT-CO2 places an extended charged side-chain in a region where each surface otherwise presents a complementary array of hydrophobic residues. Not only is this mutation sterically incompatible with this intimate subunit-subunit interaction, but the basic, polar and highly hydrophilic nature of lysine makes its accommodation in this environment extremely energetically unfavorable. It is likely that the stability of the pair of COX6C subunits in the Complex IV dimer will be affected, and their loss could render a normally protected catalytic mitochondrial-encoded subunit exposed and in direct contact with the harsh ROS-rich environment of the membrane.

Several unconfirmed Class 4 mutations have been identified in the MT-CO1 subunit of Complex IV that are associated with prostate cancer (*G6261A*, *A7158G*, *G6480A* and *T6253C*) [6], [19], [20], [58], [59]. Figure S8 highlights mutation *T6253C* which causes a M117T substitution in MT-CO1. The change from a Met to a Thr in rhodopsin protein is a known contributor to retinitis pigmentosa in humans [60]. Structural analysis of this mutation reveals that this Met side-chain is intimately involved in multiple hydrophobic interactions in the core of rhodopsin and plays a central role in its stability. Not in the core, but similarly surrounded by hydrophobic residues, M117 in Complex IV occupies a key position on the surface of MT-CO1 and interacts directly with not one, but two nuclear-encoded subunits, COX7C and COX7A1. This hydrophobic junction is likely to be critical to the correct assembly of the nuclear-encoded subunits around the core and AAS such as M117T are likely to undermine these interactions. This could result in a loss of stability, or, as depicted in Figure S8D, the complete loss of the two pairs of nuclear subunits. The precise role of COX7A1 is not yet clear, however, two recent studies in mice reveal that a COX7A1 knockout gives rise to a cardiomyopathy phenotype with reduced Complex IV activity but increased ATP production [55] while a deletion of the heart-isoform of COX7A1 impairs muscle angiogenesis and OXPHOS [61]. Interestingly, COX7C has been shown to be phosphorylated and may play a signaling role [62] and in a separate study, the expression of COX7C was identified as one of several proteins regulated in the switch between glycolysis and OXPHOS in tumor cells [63]. In either case, it would appear that loss of one or both of these subunits from Complex IV would have a direct impact on the biochemistry of the MRC and could predispose the cells towards a metabolic situation that promotes tumor growth.

From the 3D structural analysis, a further six unconfirmed Class 4 mutations are predicted as pathogenic, while only 3/7, 2/7 and 1/7 are predicted by MutationAssessor, SIFT and Polyphen, respectively (Table 3 and Figure S7). This result indicates that nuclear-mitochondrial interaction (Class 4) mutations are not readily detected by current algorithms. This is often because these sites are distal from what are considered catalytically important regions, but more significantly, these sites are subject to the co-evolution of interacting surfaces within each species [64], [65]. MRC subunit compatibility is a product of millions of years of co-evolution between nuclear and mitochondrial genomes [66] and, at least in evolutionary terms, mutations on one surface can eventually be accommodated by a compensating mutation on the interacting surface. Since current algorithms rely on conservation to identify residues of key functional importance, pathogenic mutations can be easily missed when using multiple sequence alignments from a wide range of organisms. As discussed previously, the human active site residues are often completely conserved between diverse species and even lower organisms. In stark contrast to this situation, the Class 4 residues are generally much less conserved. Even in MT-CO1, considered to be the most highly conserved of all the mitochondrial-encoded genes [67], these interacting residues often have identities of 50% or less within the reference alignment used in current tools [27]. Furthermore, lower organisms generally possess a less numerous cohort of accessory subunits, some of which are vital for regulated function in higher eukaryotes.

### Conclusion

The systematic 3D structural analysis of disease-associated mutations in Complex III and IV mtDNA protein-coding genes enabled us to generate a comprehensive visual structural mutation map and identify a novel class that currently eludes existing predictive approaches. This finding suggests that interaction region mutations play a far greater role in human diseases than previously thought and highlights the need for the bigenomic, quaternary structural organization of the mitochondrial respiratory chain to be taken into consideration when predicting the functional importance of amino-acid substitutions. 3D structural analysis provides the opportunity to visualize and predict the effect of multiple amino acid substitutions, information that could be utilized by the pharmaceutical industry to inform the development of new or improved treatments for mitochondrial diseases, perhaps on a patient specific basis in the future.

## Methods

### Data Collection

The RCSB PDB was mined for homologous MRC structures using all 13 peptide sequences from the revised Cambridge human reference mtDNA genome (NC_012920). Sequence alignments were performed using ClustalW [68] and annotated using ESPript [69] (Figure S1).

PyMOL (Schrödinger, LLC) was used to identify and display the available X-ray crystallography data for the five protein complexes of the MRC, highlighting the nuclear and mitochondrial-encoded chains. The models for each complex were derived from the following X-ray crystallographic data; Complex I (NADH dehydrogenase) 4HEA and 3M9S [2], [70], *Thermus thermophilus*; II (succinate dehydrogenase) 1ZOY [71]; *Sus scrofa*, III (cytochrome *bc*_1_) 1BE3 [8], *Bos taurus*; IV (cytochrome *c* oxidase) 1OCC [72], *Bos taurus*; V (ATP synthase) 1C17 [73], *Escherichia coli* and 1H8E, *Bos Taurus* [74].

As Complex III and IV are paradigms of how intimate nuclear-mitochondrial interactions generate complex functional machines and are highly conserved between the bovine and human homologues with sequence identities of 78, 91, 75 and 88% for MT-CYB and MT-CO1-3, respectively (Table 1), the MITOMAP database was mined for mutations in corresponding mtDNA genes with published disease associations (http://www.MITOMAP.org/MITOMAP [75]).

For a subset of mutations, individual MRC complex activity and O_2_ consumption data were collated from the literature (Tables 2). Activities were measured either polarographically or spectrophotometrically in patient muscle samples and or cybrids containing specific mutations, and are expressed relative to published mean control values. Where mean control values were unavailable, they were calculated from published control range values.

### Structural Mapping and Analysis

Ninety three point mutation sites in mtDNA Complex III and IV genes were mapped onto their corresponding bovine crystal structures based on the primary sequence alignments as a function of pathological grouping. For the subset of mutations with complex activity data, detailed analysis of the location, relative to important catalytic and/or interaction surfaces, and the potential effect of each mutation site was performed using COOT [76]. This subset was then used to define four structural classes of mutation: 1) Frameshift, 2) Active Site, 3) Binding Pocket, and 4) Nuclear-Mitochondrial Protein Interaction Region and predict the pathogenic potential of a further subset of mutations for which there was no activity data available.

### Comparison with Other Predictive Tools

The 3D structural analysis was compared with following predictive approaches: 1) the recently published integrated approach that uses SIFT (http://sift.jcvi.org/www/SIFT_related_seqs_submit.html) and PolyPhen (http://genetics.bwh.harvard.edu/pph2) programs [27] and 2) the MutationAssessor program (http://mutationassessor.org/v1/) [77] (Tables 2 and 3). SIFT predictions were obtained by submitting the human MT-CO1-3 and MT-CYB sequences along with the 10 species recommended by the integrated approach: *Homo sapiens, Bos taurus, Mus musculus, Rattus norvegicus, Gallus gallus, Xenopus laevis, Drosophila melanogaster, Strongylocentrotus droebachiensis and Caenorhabditis elegans*, and the substitutions of interest. SIFT then generated an alignment and assigned a score to each substitution ranging from zero to one. Substitutions with a SIFT score of <0.05 were predicted to affect protein function, while those >0.05 were predicted as tolerant (not likely to affect protein function). Polyphen predictions were also obtained by submitting the four human mitochondrial protein sequences along with the substitutions of interest. Polyphen then assigned scores to each substitution, ranging from zero to a positive number, using protein sequences from the UniProtKB/UniRef100 Release 2011_12 (14-Dec-2011), structures from the PDB/DSSP Snapshot 03-Jan-2012 (78,304 entries) and multiple alignments (UCSC MultiZ) of 45 vertebrate genomes with hg19/GRCh37 human genome (08-Oct-2009). Substitutions with a score of zero were predicted as benign, while substitutions with a positive number were predicted as pathogenic. MutationAssessor predictions were obtained by submitting the Uniprot protein accession numbers for the four human mitochondrial proteins and the substitutions of interest. MutationAssessor then generated an alignment using Uniprot and Refseq protein sequences and assigned a score to each substitution. MutationAssessor predicted substitutions with a score of >3.5, 3.5–1.9, 1.8–0.8 and, <0.8 as high impact, medium impact, low impact and neutral, respectively.

## Supporting Information

Figure S1 **Sequence alignment of human** ***vs*** **bovine complexes used in the 3D structural analysis of amino acid substitutions in Complex III and IV mitochondrial-encoded subunits, shown in** **Table 2** **and** **3** **.**
(PDF)

Figure S2 **3D structural analysis flowchart used for the classification and pathogenic prediction of mutations in the mtDNA complex III and IV genes (** ***mt-cyb and mt-co1-3*** **, respectively) reported with human disease associations, with and without known biochemical effects, see** **Table 2** **and** **3** **.**
(PDF)

Figure S3 **Structural consequences of a frameshift mutation, further details in** **Table 2** **.**
(PDF)

Figure S4 **The position of 10 individual mutations found within an active site region of MT-CO1 and a binding pocket of MT-CYB of Complex IV and III, respectively, details in** **Table 2** **and** **3** **.**
(PDF)

Figure S5 **Detailed view of active site mutations I280T and V380I, further information on predicted pathogenicity can be found in** **Table 2** **and** **3** **.**
(PDF)

Figure S6 **Substrate-binding cavity mutation S35P, further details on their biochemistry and predicted pathogenicity can be found in** **Table 2** **.**
(PDF)

Figure S7 **Interaction region (Class 4) mutations in mitochondrial-encoded complex IV genes (** ***mt-co1-2*** **), details in** **Table 2** **and** **3** **.**
(PDF)

Figure S8 **Prediction of dramatic consequences in assembly and stability of an interaction region mutation, more details can be found in** **Table 3** **.**
(PDF)
