# Supplementary material for: Structural Analysis of Mitochondrial Mutations Reveals a Role for Bigenomic Protein Interactions in Human Disease
Source: PLoS One. 2013 Jul 9;8(7):e69003. doi: 10.1371/journal.pone.0069003 (PMC3706435; doi:10.1371/journal.pone.0069003)
Supplement: Figure S1 — Sequence alignment of human vs bovine complexes used in the 3D structural analysis of amino acid substitutions in Complex III and IV mitochondrial-encoded subunits, shown in Table 2 and 3 . (PDF) [file pone.0069003.s001.pdf]

Figure S1. Sequence alignment of human *vs* bovine complexes.

Complex III

MT-CYB

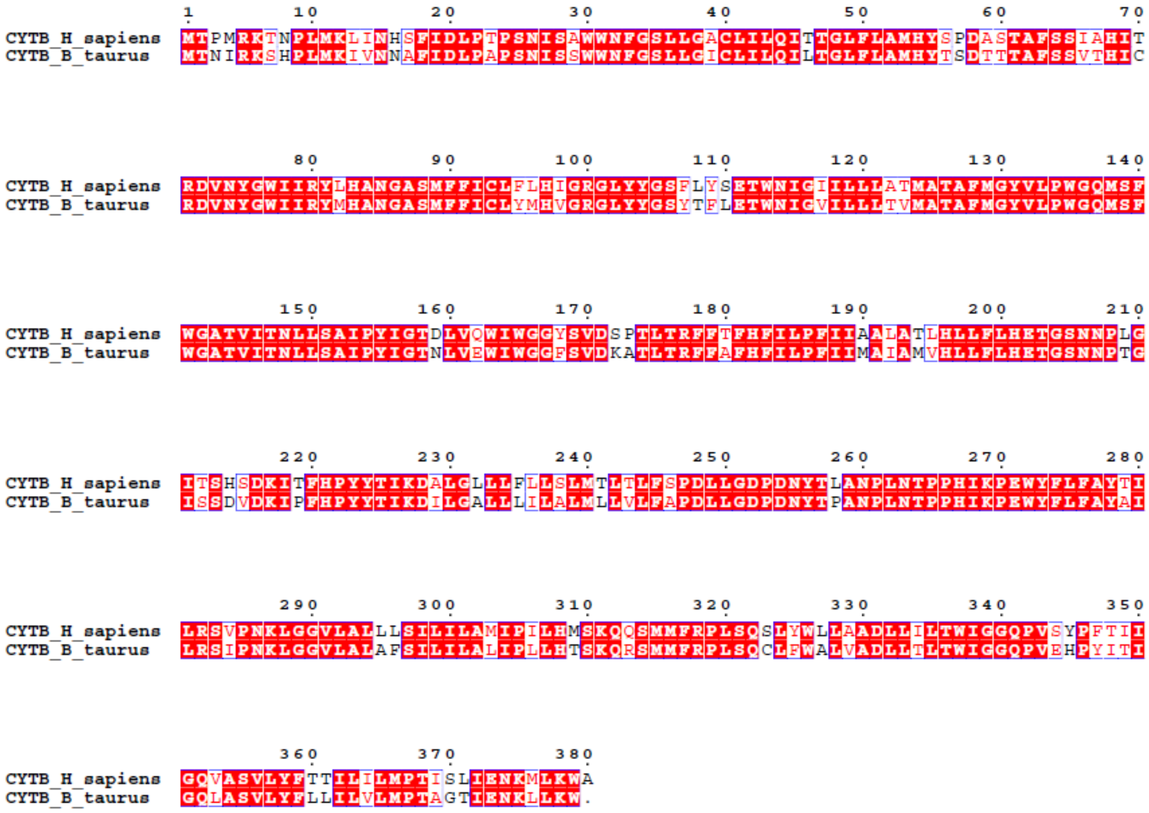

Complex IV

a) MT-CO1

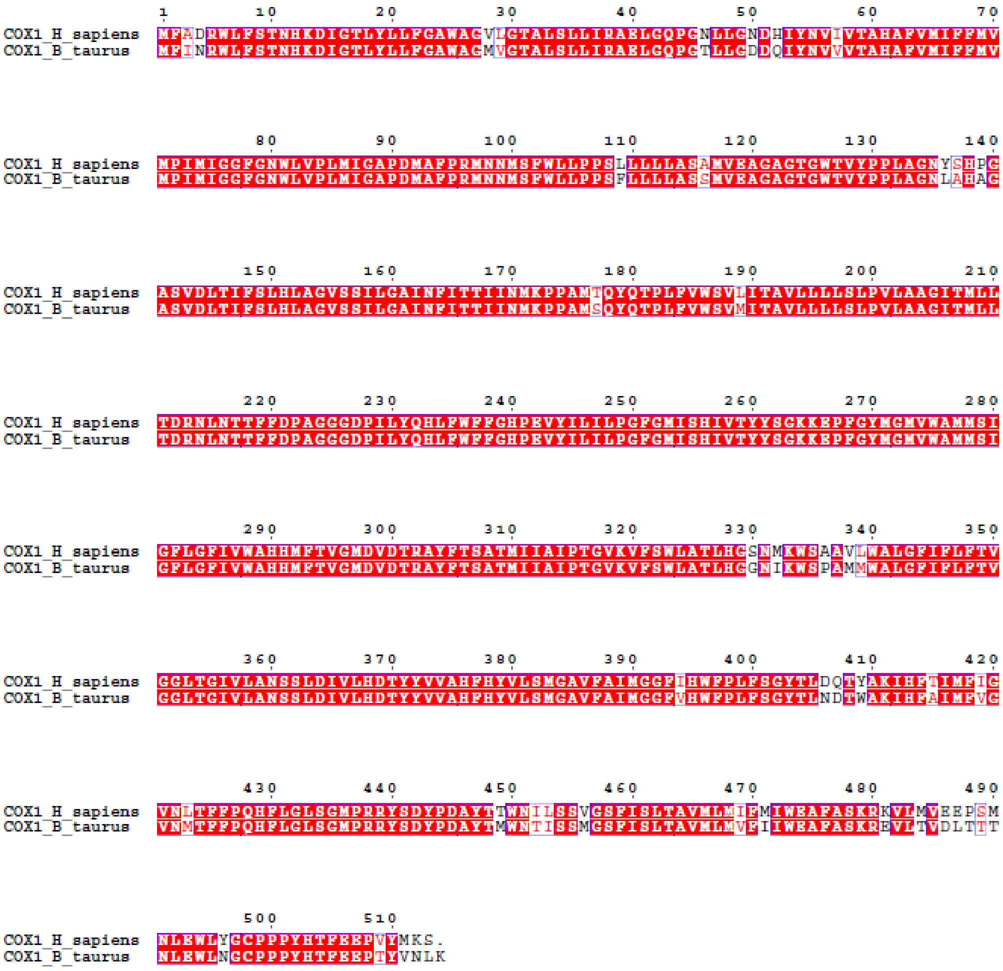

b) MT-CO2

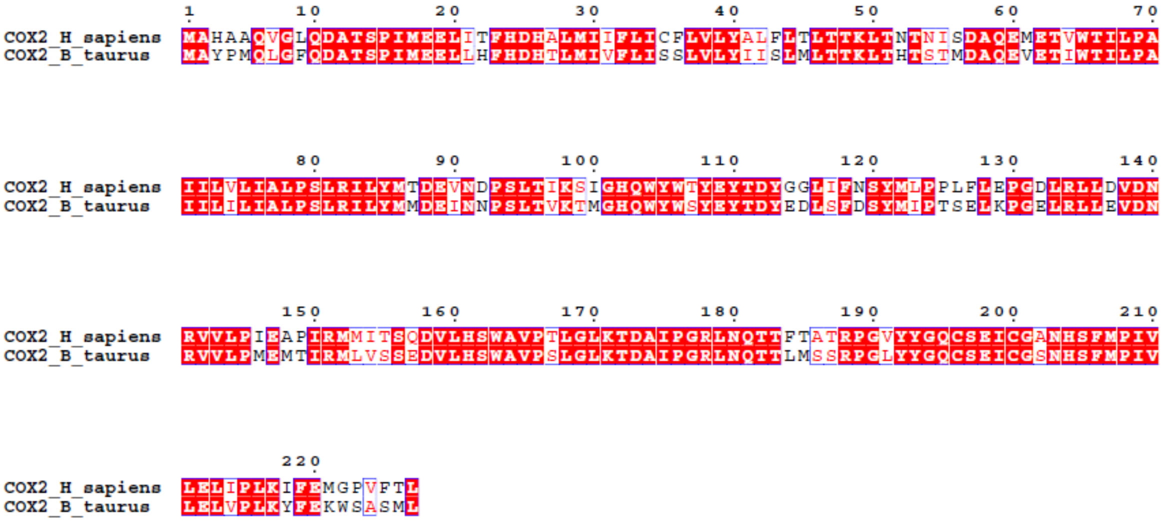

c) MT-CO3

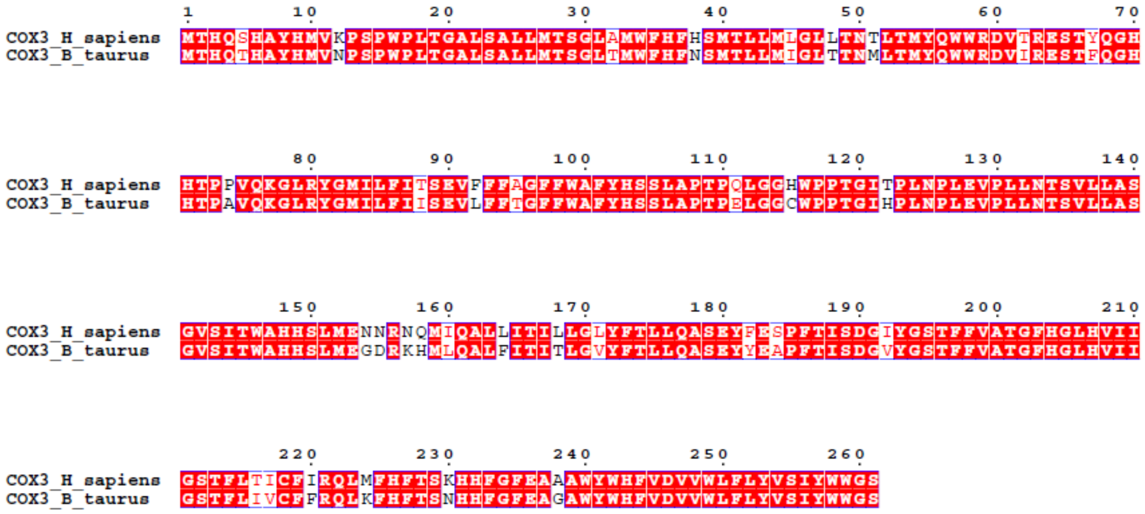
1 10 20 30 40 50 60 70
COX3_H_sapiens MTHQSHAYHMVKPSPWPLTGALSALLMTSGLAMWFHFHSMTLLMLGLLTNTLTMYQWWRDVTRESTYQGH
COX3_B_taurus MTHQTHAYHMVNPSPWPLTGALSALLMTSGLTMWFHFNSMTLLMIGLTTNMLTMYQWWRDVIRESTFQGH
80 90 100 110 120 130 140
COX3_H_sapiens HTPPVQKGLRYGMILFITSEVFFFAGFFWAFYHSSLAPTPQLGGHWPPTGITPLNPLEVPLLNTSVLLAS
COX3_B_taurus HTPAVQKGLRYGMILFIISEVLFFTGFFWAFYHSSLAPTPELGGCWPPTGIHPLNPLEVPLLNTSVLLAS
150 160 170 180 190 200 210
COX3_H_sapiens GVSITWAHHSLMENNRNQMIQALLITILLGLYFTLLQASEYFESPFTISDGIYGSTFFVATGFHGLHVII
COX3_B_taurus GVSITWAHHSLMEGDRKHMLQALFITITLGVYFTLLQASEYYEAPFTISDGVYGSTFFVATGFHGLHVII
220 230 240 250 260
COX3_H_sapiens GSTFLTICFIRQLMFHFTSKHHFGFEAAAWYWHFVDVVWLFLYVSIYWWGS
COX3_B_taurus GSTFLIVCFFRQLKFHFTSNHHFGFEAGAWYWHFVDVVWLFLYVSIYWWGS
