# Supplementary material for: Structural Analysis of Mitochondrial Mutations Reveals a Role for Bigenomic Protein Interactions in Human Disease
Source: PLoS One. 2013 Jul 9;8(7):e69003. doi: 10.1371/journal.pone.0069003 (PMC3706435; doi:10.1371/journal.pone.0069003)

Figure S2. 3D structural analysis flowchart. The tool allows the classification and functional effects any mtDNA mutation in Complex III and IV genes to be predicted, even if biochemical data from a patient sample is absent.

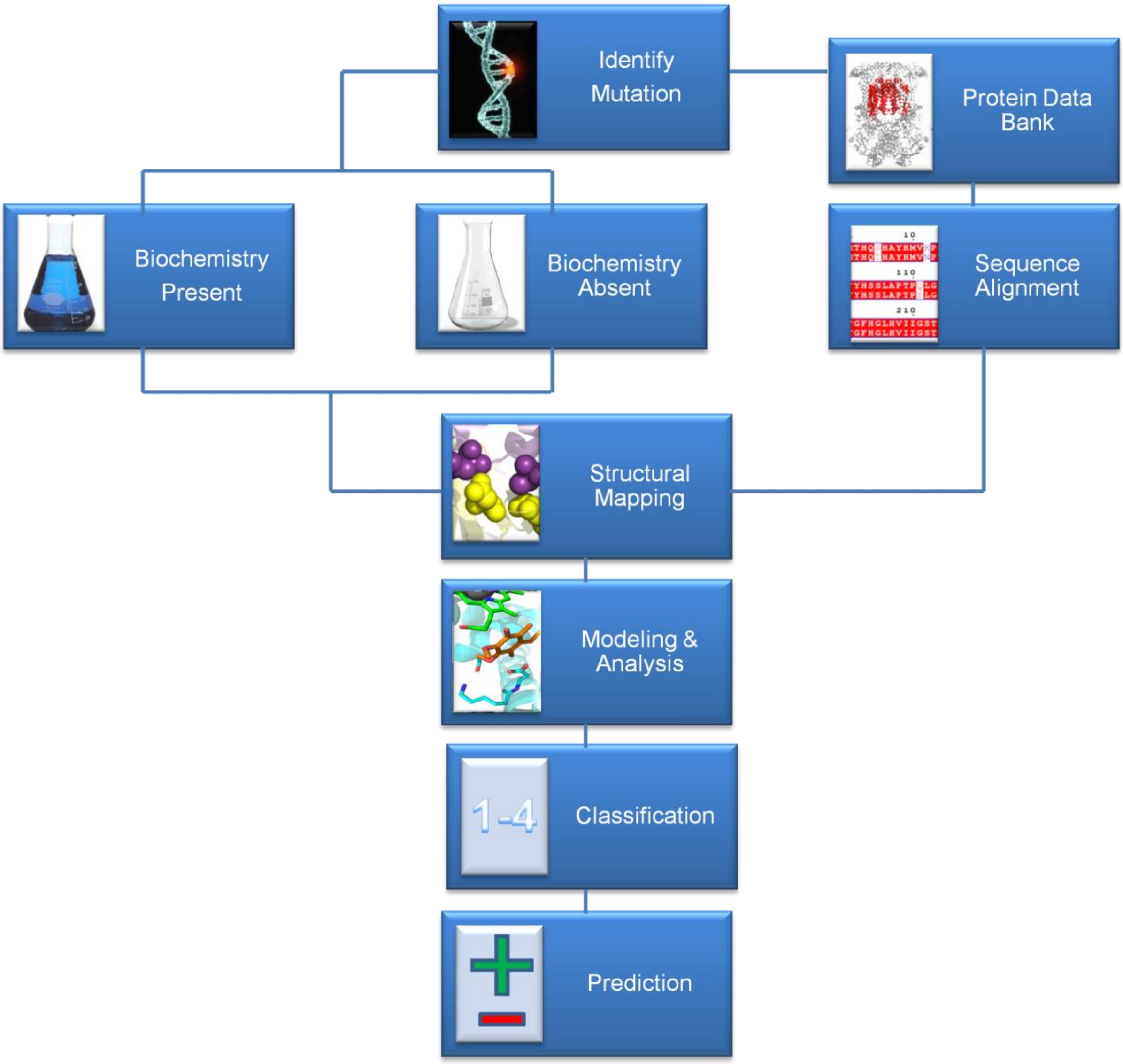

Supplement: Figure S2 — 3D structural analysis flowchart used for the classification and pathogenic prediction of mutations in the mtDNA complex III and IV genes ( mt-cyb and mt-co1-3 , respectively) reported with human disease associations, with and without known biochemical effects, see Table 2 and 3 . (PDF) [file pone.0069003.s002.pdf]
