# Supplementary material for: Structural Analysis of Mitochondrial Mutations Reveals a Role for Bigenomic Protein Interactions in Human Disease
Source: PLoS One. 2013 Jul 9;8(7):e69003. doi: 10.1371/journal.pone.0069003 (PMC3706435; doi:10.1371/journal.pone.0069003)

Figure S3. Structural consequences of a frameshift mutation. Complex IV is shown with nuclear-encoded subunits in grey, MT-CO2 subunits in yellow and MT-CO3 subunits in green. MT-CO1 is rendered as a space-filling model colored in orange, highlighting its central position within each monomer. (A) Wild-type. (B) The *6020del5* deletion associated with Motor Neuron Disease changes four amino acids and generates a stop codon at amino acid position 39 that deletes over 90% of the MT-CO1 polypeptide chain, shown as the comparative loss of protein between models. Rotation of the wild-type (C) and deleted molecule (D) by 90 degrees relative to the membrane reveals how the deletion affects the interaction of multiple subunits that surround MT-CO2.

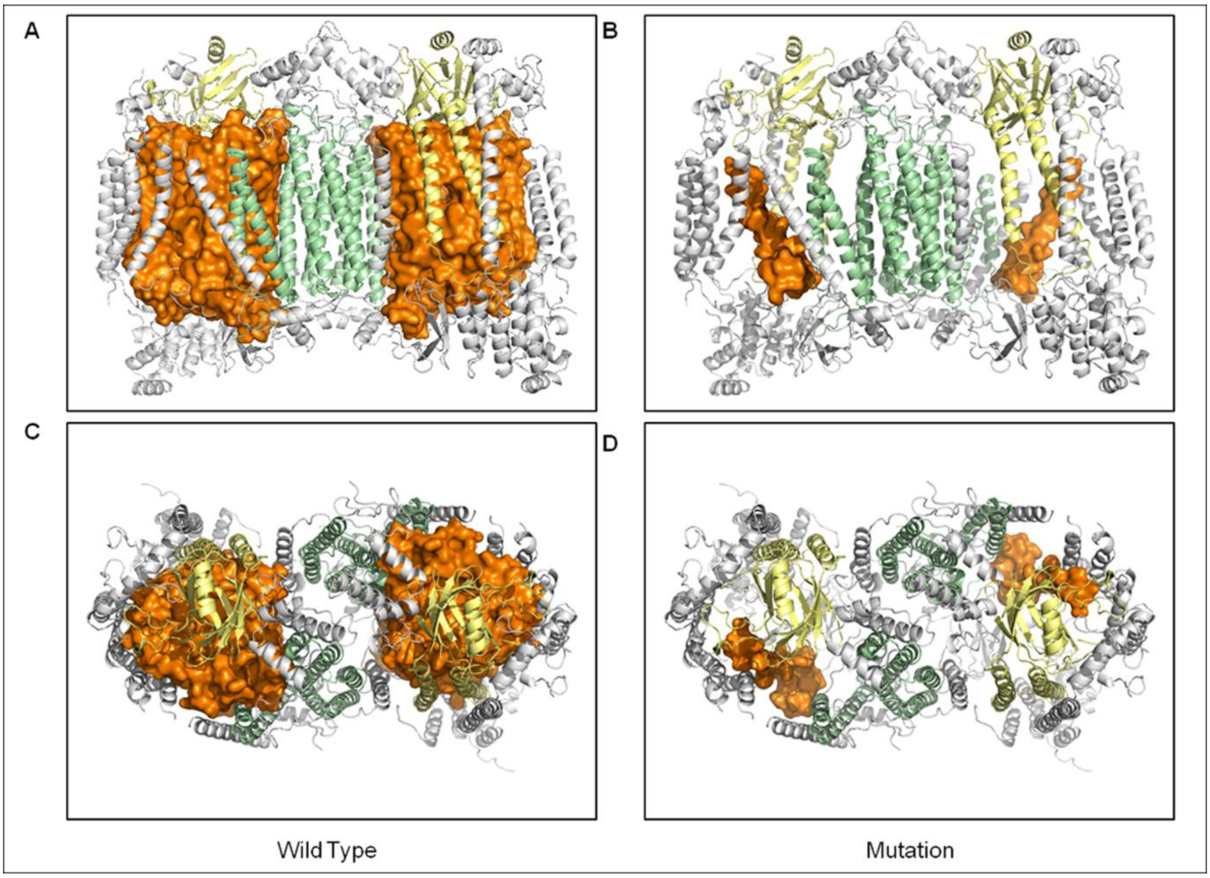

Supplement: Figure S3 — Structural consequences of a frameshift mutation, further details in Table 2 . (PDF) [file pone.0069003.s003.pdf]
