# Supplementary material for: Structural Analysis of Mitochondrial Mutations Reveals a Role for Bigenomic Protein Interactions in Human Disease
Source: PLoS One. 2013 Jul 9;8(7):e69003. doi: 10.1371/journal.pone.0069003 (PMC3706435; doi:10.1371/journal.pone.0069003)

Figure S4. The position of 10 individual mutations found within an active site region of MT-CO1 (Left) and a binding pocket of MT-CYB (Right) of Complex IV and III, respectively. The mutations are shown as side-chains rendered as spheres and coloured according to their associated disease (see Figure 2 legend). Heme groups are shown as green stick models with bound substrate ubiquinone molecule in red (PDB 1NTZ). Multiple mutation sites clustering around, and directly interacting with, the heme group *a* and those corresponding to the side-chains that make direct contributions to the ubiquinone binding pocket are shown. *T6721C* (M273T), *T6742C* (I280T), *G6955A* (G352D), *T14849C* (S35P), *G14846A* (G34S), *A15579G* (Y278C) and *G15762A* (G339E) are confirmed mutations, while *G7041A* (V380I), *G7023A* (V374M) and *A14841G* (N32S) are unconfirmed (see main text for definitions).

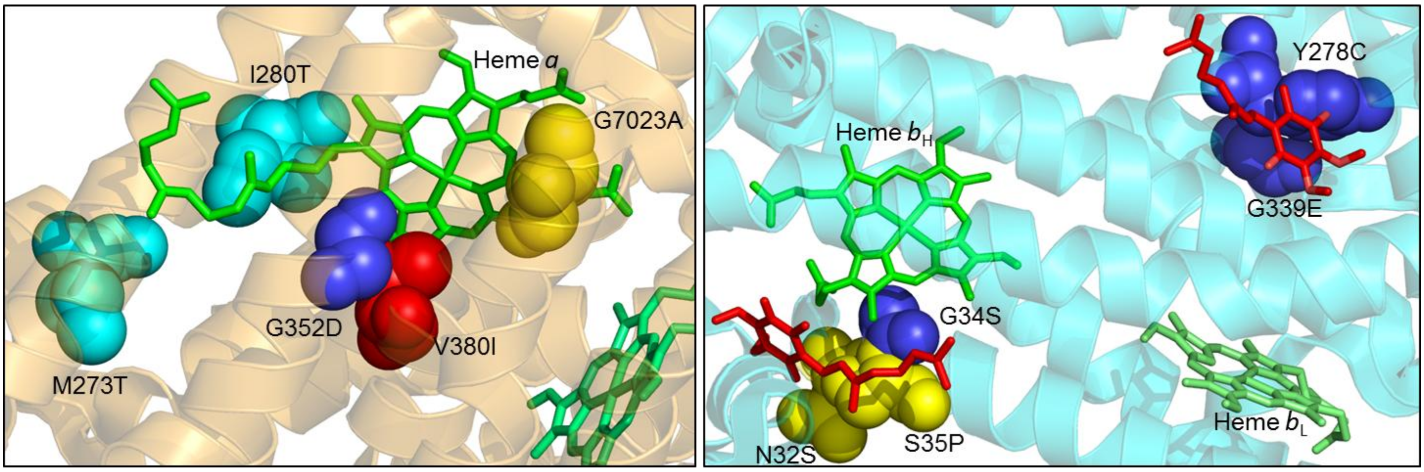

Supplement: Figure S4 — The position of 10 individual mutations found within an active site region of MT-CO1 and a binding pocket of MT-CYB of Complex IV and III, respectively, details in Table 2 and 3 . (PDF) [file pone.0069003.s004.pdf]
