# Supplementary material for: Structural Analysis of Mitochondrial Mutations Reveals a Role for Bigenomic Protein Interactions in Human Disease
Source: PLoS One. 2013 Jul 9;8(7):e69003. doi: 10.1371/journal.pone.0069003 (PMC3706435; doi:10.1371/journal.pone.0069003)

Figure S5. Detailed view of active site mutations I280T and V380I. The active site region of the MT-CO1 subunit of complex IV is depicted as a ribbon diagram in orange. The heme a_3 is colored in green with the Fe atom in grey. (A) The wild-type I280 side-chain is depicted as Van der Waals spheres and can be compared to (B) where the oxygen of the mutated residue, T280 is colored red. (C) The position of the wild-type V380 to the central Fe of heme a_3. (D) The steric clash associated with the mutation to I380 due to the extension of the side chain towards the central heme Fe atom.

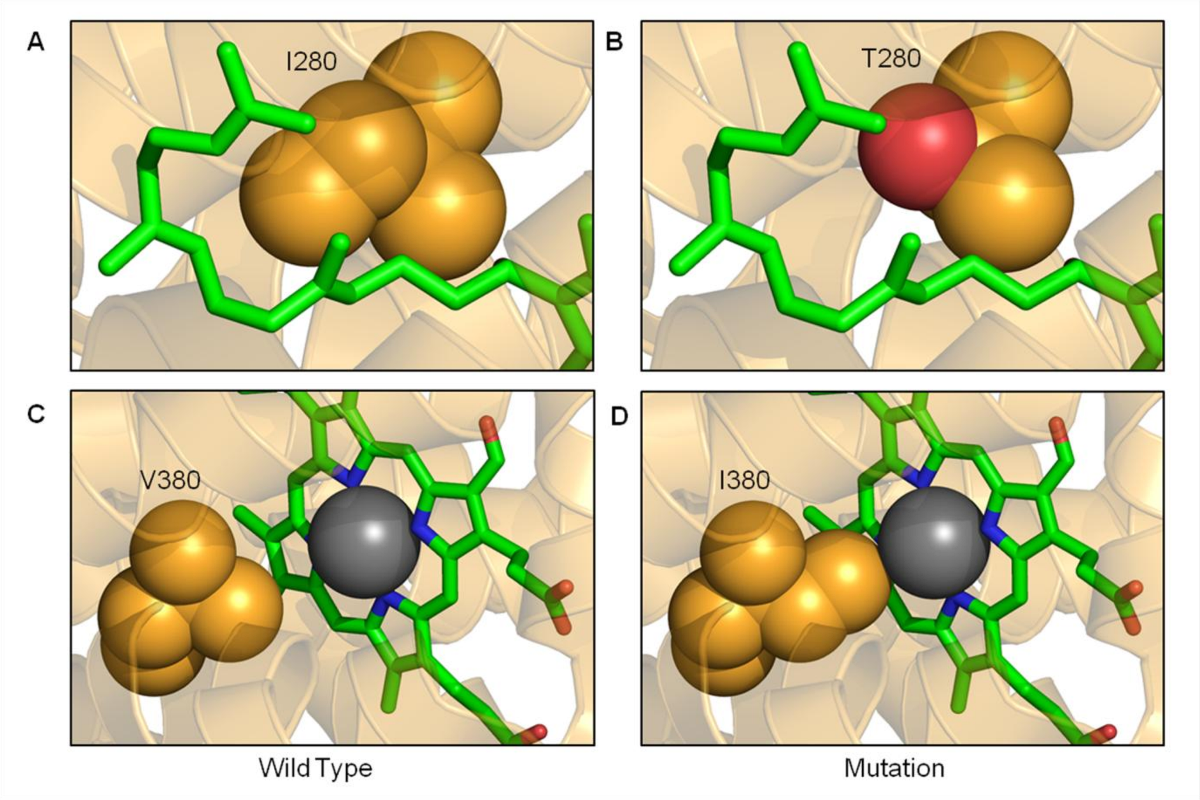

Supplement: Figure S5 — Detailed view of active site mutations I280T and V380I, further information on predicted pathogenicity can be found in Table 2 and 3 . (PDF) [file pone.0069003.s005.pdf]
