# Supplementary material for: Structural Analysis of Mitochondrial Mutations Reveals a Role for Bigenomic Protein Interactions in Human Disease
Source: PLoS One. 2013 Jul 9;8(7):e69003. doi: 10.1371/journal.pone.0069003 (PMC3706435; doi:10.1371/journal.pone.0069003)

Figure S6. Substrate-binding cavitiy mutation S35P. (A) The position of ubiquinone, the natural substrate, is shown in orange within the Q_i site of MT-CYB (blue ribbon) in relation to the heme (green) to the wild-type residue S35. (B) The consequence of the mutation P35 is depicted in red, showing the likely region of helix disorder caused by the inflexible nature of the proline side-chain. The same area is illustrated as a solvent accessible surface model with the position of S35 shown in dark blue (C) and the area of disruption caused by P35 shown in red (D).

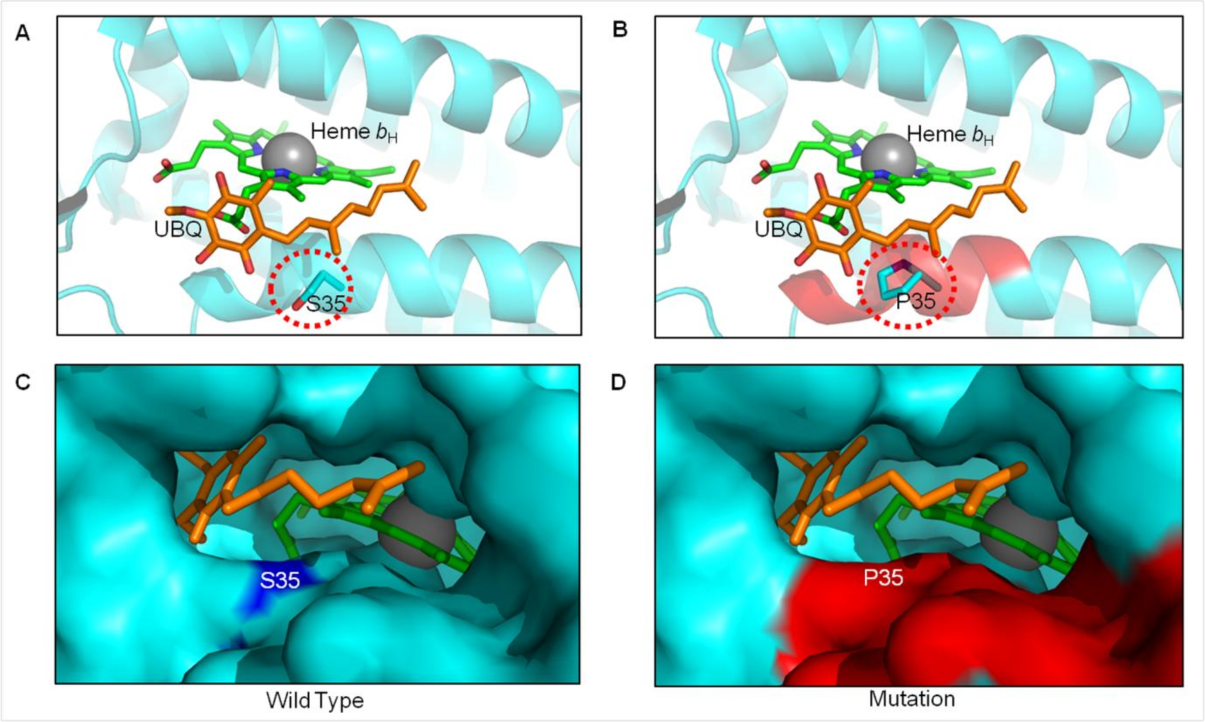

Supplement: Figure S6 — Substrate-binding cavity mutation S35P, further details on their biochemistry and predicted pathogenicity can be found in Table 2 . (PDF) [file pone.0069003.s006.pdf]
