# Supplementary material for: Structural Analysis of Mitochondrial Mutations Reveals a Role for Bigenomic Protein Interactions in Human Disease
Source: PLoS One. 2013 Jul 9;8(7):e69003. doi: 10.1371/journal.pone.0069003 (PMC3706435; doi:10.1371/journal.pone.0069003)

Figure S7. Interaction region (Class 4) mutations in mitochondrial-encoded complex IV genes (MT-CO1-2). Mitochondrial-encoded mutation sites are highlighted as previous (see Figure 2 legend), with the relevant nuclear-encoded residue and subunit in purple or pink. Top, left to right: MT-CO2 *G7637A* (E18K) and *T7671A* (M29K) to subunit COX6C residues Y47 and Y35 respectively, MT-CO2 *G7859A* (D92N) and *C7868T* (L95F) to subunit COX6B1 residues F16 and T13 respectively, and MT-CO1 *G6261A* (A120T) to subunit COX7C residue Q43. Bottom, left to right: MT-CO1 *A7158G* (I419V) to subunit COX4 residue I89, MT-CO1 *G6480A* (V193I) to subunit COX6A2 residue A4 and MT-CO1 *T6253C* (M117T) interacts with two nuclear-encoded subunits, COX7C residue H42 (purple) and COX7A1 residue S54 (pink), respectively. All mutations, apart from *T7671A*, are unconfirmed mutations (for definitions, see the main text).

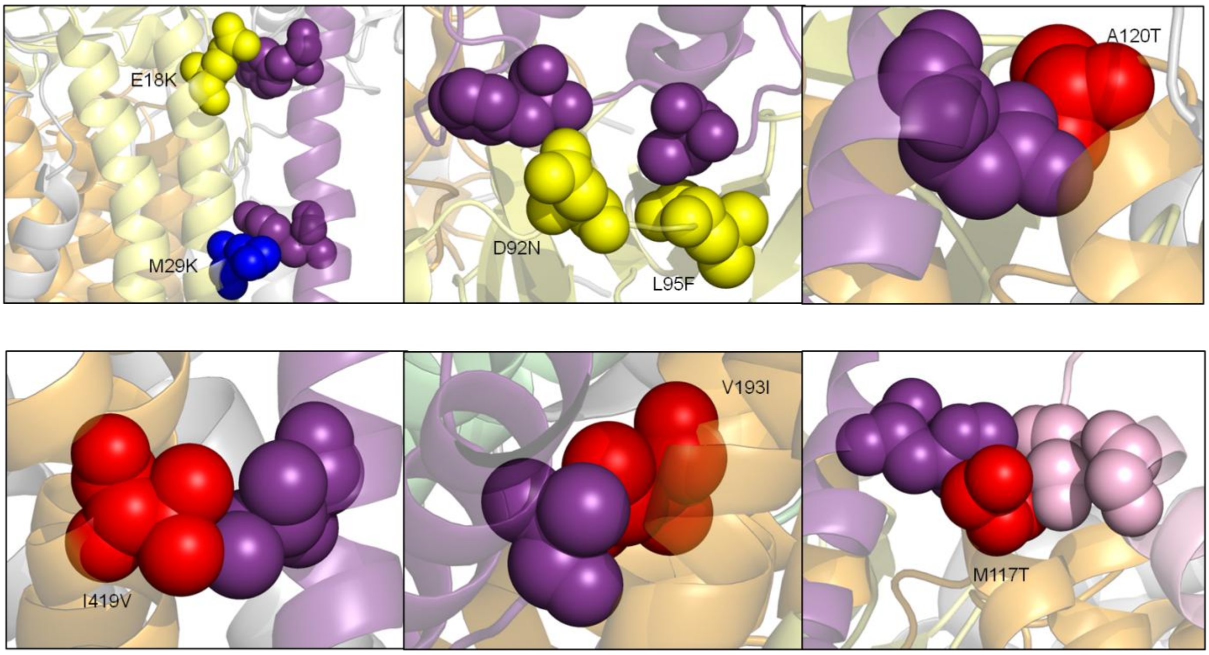

Supplement: Figure S7 — Interaction region (Class 4) mutations in mitochondrial-encoded complex IV genes ( mt-co1-2 ), details in Table 2 and 3 . (PDF) [file pone.0069003.s007.pdf]
