# Supplementary material for: Structural Analysis of Mitochondrial Mutations Reveals a Role for Bigenomic Protein Interactions in Human Disease
Source: PLoS One. 2013 Jul 9;8(7):e69003. doi: 10.1371/journal.pone.0069003 (PMC3706435; doi:10.1371/journal.pone.0069003)

Figure S8. Prediction of dramatic consequences in assembly and stability of an interaction mutation. (A) A ribbon diagram of Complex IV depicts the interface between MT-CO1 (orange) and the two nuclear-encoded surface subunits, 7C (left, pink) and 7A1 (right, purple). The wild type residue M177 is shown in stick representation (orange with sulfur the atom in green) in relation to neighboring residues H42 from subunit 7C and residues S54 and F55 from subunit 7A1. (B) The mutation T117 is shown in the same color scheme, resulting in a loss of the sulfur atom and gain of the terminal oxygen (red). (C) The location of the wild type residue M117 is highlighted on a surface representation of Complex IV with a dotted red circle. (D) A surface model depicting the loss of subunits 7C (pink) and 7A1 (purple) as a result of the structural incompatibility of the MT-CO1 T117 mutation.

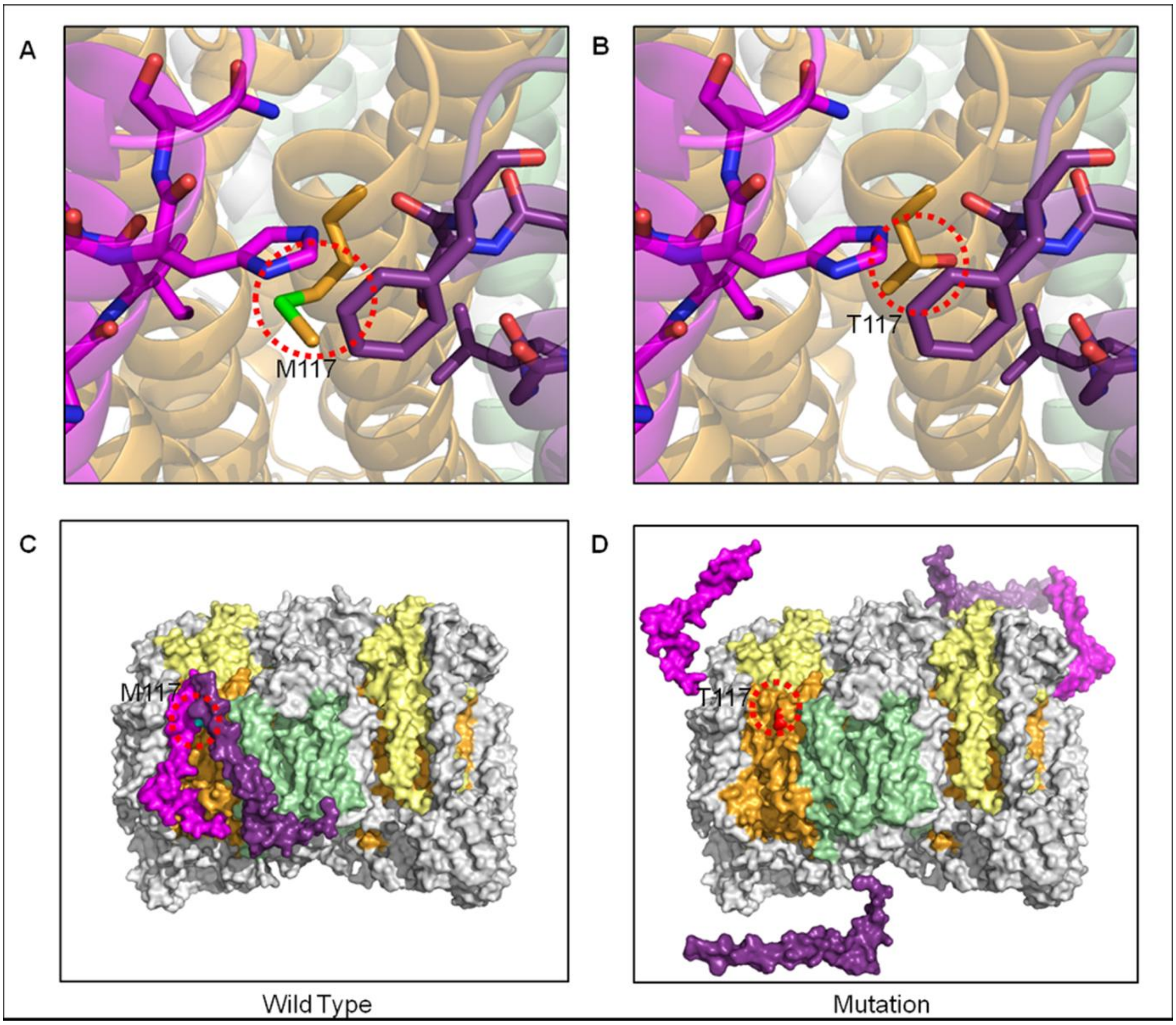

Supplement: Figure S8 — Prediction of dramatic consequences in assembly and stability of an interaction region mutation, more details can be found in Table 3 . (PDF) [file pone.0069003.s008.pdf]
